# Bisphosphonates and risk of atrial fibrillation: a meta-analysis

**DOI:** 10.1186/ar2938

**Published:** 2010-02-19

**Authors:** Seo Young Kim, Min Jung Kim, Suzanne M Cadarette, Daniel H Solomon

**Affiliations:** 1 Division of Rheumatology, Allergy and Immunology, Department of Medicine, Brigham and Women's Hospital, 75 Francis Street, Boston, MA, 02115, USA; 2 Division of Pharmacoepidemiology, Department of Medicine, Brigham and Women's Hospital, 75 Francis Street, Boston, MA, 02115, USA; 3 Division of Nuclear Medicine and Molecular Imaging, Department of Radiology, Brigham and Women's Hospital, 75 Francis Street, Boston, MA, 02115, USA; 4 Leslie Dan Faculty of Pharmacy, University of Toronto, 144 College Street, Toronto, Ontario, M5S 3M2, Canada

## Abstract

**Introduction:**

Bisphosphonates are the most commonly used drugs for the prevention and treatment of osteoporosis. Although a recent FDA review of the results of clinical trials reported no clear link between bisphosphonates and serious or non-serious atrial fibrillation (AF), some epidemiologic studies have suggested an association between AF and bisphosphonates.

**Methods:**

We conducted a meta-analysis of non-experimental studies to evaluate the risk of AF associated with bisphosphonates. Studies were identified by searching MEDLINE and EMBASE using a combination of the Medical Subject Headings and keywords. Our search was limited to English language articles. The pooled estimates of odds ratios (OR) as a measure of effect size were calculated using a random effects model.

**Results:**

Seven eligible studies with 266,761 patients were identified: three cohort, three case-control, and one self-controlled case series. Bisphosphonate exposure was not associated with an increased risk of AF [pooled multivariate OR 1.04, 95% confidence interval (CI) 0.92-1.16] after adjusting for known risk factors. Moderate heterogeneity was noted (I-squared score = 62.8%). Stratified analyses by study design, cohort versus case-control studies, yielded similar results. Egger's and Begg's tests did not suggest an evidence of publication bias (*P* = 0.90, 1.00 respectively). No clear asymmetry was observed in the funnel plot analysis. Few studies compared risk between bisphosphonates or by dosing.

**Conclusions:**

Our study did not find an association between bisphosphonate exposure and AF. This finding is consistent with the FDA's statement.

## Introduction

Osteoporosis is a major public health threat for an estimated 44 million Americans or 55% of the population 50 years of age and older [1]. There were approximately two million new fractures in the US in 2005 with a direct medical cost of US$17 billion [2]. Bisphosphonates are the main therapy for postmenopausal and glucocorticoid-induced osteoporosis. Currently, four oral or intravenous forms of bisphosphonates (alendronate, risedronate, ibandronate, and zoledronic acid) are approved for the treatment of osteoporosis in the US. Known side effects of bisphosphonates include gastrointestinal mucosal irritation, such as esophageal ulcer, bone pain, muscle cramps or pain, hyperphosphatemia, hypocalcemia, and rarely osteonecrosis of the jaw [3,4].

An increased risk of atrial fibrillation (AF) has been noted with use of bisphosphonates, especially with intravenous zoledronic acid [5-7]. AF is the most common arrhythmia among older adults. Common risk factors include older age, male gender, dilated cardiomyopathy, diabetes, hypertension, coronary artery disease, obstructive sleep apnea, cardiac surgery, high-dose corticosteroid therapy, alcohol excess, and alcohol withdrawal [8-10]. AF contributes to approximately 35% of strokes in an octogenarian population, increases the overall risk of stroke by five-fold, and is associated with particularly severe strokes [11]. With the aging population, AF and its consequences will become an increasingly common medical and economic problem.

In October 2007, the US Food and Drug Administration (FDA) first announced that the agency was assessing the potential risk of AF associated with bisphosphonate use [12]. After reviewing data from spontaneous post-marketing reports and clinical trials of bisphosphonates, the FDA stated that healthcare providers and patients should not change either their prescribing practices or their use of bisphosphonates [12]. A year later, the FDA released updated information based on further information from four placebo-controlled clinical trials and concluded that there was no clear association between bisphosphonate exposure and the rate of serious or non-serious AF events [13]. At that time, the FDA stated that that they would explore the feasibility of conducting additional epidemiologic studies to further examine the issue. Subsequently, a number of large population-based studies examining the risk of AF with bisphosphonates have been published [7,14-19]. The results from two meta-analytic studies [20,21] are also available but information one could draw from these studies are somewhat limited owing to a small study size and significant heterogeneity among the included studies. Population-based epidemiologic studies offer several advantageous over clinical trial data, particularly to study unexpected and rare adverse reactions such as AF in bisphosphonate users (incidence rate of AF is less than 2 per 100 person-year [14,17]). Clinical trials are often underpowered to detect rare adverse events and lack of generalizability owing to specific inclusion and exclusion criteria [22]. Given such potential limitations of clinical trials and meta-analyses of clinical trials, we therefore conducted a systematic review and meta-analysis of non-experimental studies to determine the association between the use of bisphosphonates and the risk of AF or flutter.

## Materials and methods

### Data sources

**Figure 1:**
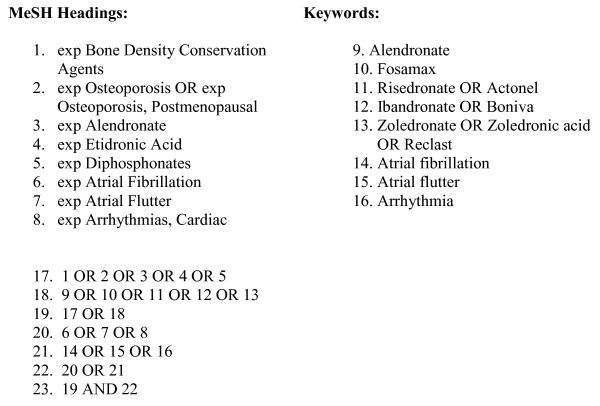
**Search strategy**.

We searched two major electronic databases -- MEDLINE (1981 to September 2009), EMBASE (1981 to September 2009) -- for studies of the association between AF and the use of bisphosphonates in patients with osteoporosis. We also searched abstracts from scientific meetings (American College of Rheumatology and International Conference on Pharmacoepidemiology and Therapeutic Risk Management) and bibliographies of identified reports and review articles for additional references. We imposed no geographic restrictions. Our search strategy is described in Figure 1. Only studies published in English were included.

### Study eligibility and selection

To be eligible for inclusion, we only considered population-based non-experimental studies of adult patients, studies reporting the risk of AF or flutter associated with bisphosphonate use, and studies examining the effects of bisphosphonates for the prevention or treatment of osteoporosis or fractures. Studies of patients with malignancy such as breast or prostate cancer were excluded. Two authors (SYK and MJK) independently screened each of the potential titles, abstracts, and/or full-texts to determine if they were eligible for inclusion. Areas of disagreement or uncertainty were resolved by consensus.

### Data abstraction and quality assessment

All data were independently abstracted in duplicate by two authors (SYK and MJK) using a data abstraction form. Data on the study characteristics, such as author name, year of publication, country, sample size, mean age of patients, type of bisphosphonate used, and number of outcome, unadjusted and multivariate, or adjusted, and risk of AF, were collected. The Newcastle-Ottawa Scale was used to assess the quality of studies [23] (Tables 1 and 2). A quality score was calculated on the basis of three major components: selection of the study groups (0 to 4 points), quality of the adjustment for confounding (0 to 2 points) and ascertainment of the outcome of interest in the cohorts (0 to 3 points). A higher score represents better methodological quality. Adjustment for known AF risk factors, duration of follow up of at least one year for cohort studies, and adequate outcome ascertainment were criteria of higher quality.

**Table 1:** Summary of included cohort studies and characteristics

Study, year, country	Subjects	Mean age (years)	Mean follow-up (years)	Type of bisphosphonate	AF definition	Total AF events (n)	Variables matched and/or controlled	Quality assessment score^§^
Bunch and colleagues [15], 2009, USA	9,623 (98 BIS users and 9,525 non-users) in angiographic research database	60	4.1	Alendronate Etidronate Ibandronate Risedronate Zoledronic acid	Hospital discharge ICD-9 codes and EKG data	974	Age, HTN, hyperlipidemia, DM, HF, CAD, etc	4/2/3
	37,485 (7,489 BIS users and 29,996 non-users) in a commercial health care system database	51	4.6			1012		

Abrahamsen and colleagues [14], 2009, Denmark	43,033 patients (14,302 BIS users and 28,731 non-users) with fractures in the National Hospital Discharge register	74	2.7	Alendronate Clodronate Etidronate Ibandronate Risedronate	Prescription for a cardiac glycoside +/- ICD-10 codes	797 *	Age, gender, fracture sites, medications use for DM, HTN, hypercholesterolemia, and thrombosis, and Charlson index	4/2/3

Huang and colleagues [17], 2009, Taiwan	27,257 women (21,037 BIS users and 6,220 raloxifene users) with osteoporosis in the National Health Insurance database	74	0.6	Alendronate	Inpatient or outpatient ICD-9 codes	821	DM, CAD, COPD, history of AF, HTN, and total defined daily dose of drugs	3/2/2

* only in BIS users, ^§ ^Methodologic quality assessment score based on Newcastle-Ottawa Scale [23]; selection (0 to 4), comparability (0 to 2), and outcome score (0 to 3) points with a higher score representing a better quality.

AF, atrial fibrillation; BIS, bisphosphonates; CAD, coronary artery disease; COPD, chronic obstructive pulmonary disease; DM, diabetes mellitus; EKG, electrocardiogram; HF, heart failure; HTN, hypertension; ICD, International Classification of Diseases.

**Table 2:** Summary of included case-control studies and characteristics

Study, Year, Country	Subjects	Mean age (year)	Mean duration (year)	Type of bisphosphonate	AF definition	Total AF events (n)	Variables matched and/or controlled	Quality assessment score^§^
Heckbert and colleagues [7], 2008, USA	1,685 women in a commercial health care system database	73	20	Alendronate	ICD-9 codes verified by medical records +/- EKG	719	Age, HTN, calendar year, osteoporosis, and any cardiovascular disease	4/2/3
Sorensen and colleagues [19], 2008, Denmark	81,640 women in the National Registry of Patients	>75	Not reported	Alendronate Etidronate	ICD-8 and 10 codes	13586	Age, county, cardiovascular disease, renal failure, DM, HRT, fracture, cancer, alcoholism, pulmonary disease, anti-thyroid drugs, osteoporosis, steroid use, etc	3/2/3
Grosso and colleagues* [16], 2009, UK	2,195 women with AF in the UK General Practice Research Database	82	13	Alendronate Risedronate	Diagnosis codes and Read codes	2195	Age^+^	3/2/3
Levesque and colleagues [18], 2009, Canada	63,843 adults (BIS users and raloxifene users) in the Quebec health database	75	4	Alendronate Etidronate^a^ Risedronate^a^	Not reported	2149	Age, cohort entry year, follow-up, CAD, HTN, and use of HRT	N/A

AF, atrial fibrillation; BIS, bisphosphonates; CAD, coronary artery disease; DM, diabetes mellitus; EKG, electrocardiogram; HRT, hormone replacement therapy; HTN, hypertension; ICD, International Classification of Diseases.

* self-controlled case-series. ^+ ^no other adjustment not needed in self-controlled study, N/A, not applicable (only the abstract was available at the time of this study), ^a ^risks specific to etidronate or risedronate not used in meta-analysis, ^§ ^Methodologic quality assessment score based on Newcastle-Ottawa Scale [23]; selection (0 to 4), comparability (0 to 2), and outcome score (0 to 3) points with a higher score representing a better quality.

### Statistical analysis

We chose to use odds ratio (OR) as a measure of effect size because it is one of the most commonly used effect measures for observational studies [24]. We assumed that either relative risk or hazard ratio was equivalent to OR as the probability of the outcome, AF, was expected to be low [25]. Pooled estimates of multivariate ORs were calculated using the DerSimonian and Laird random effects model [26,27] for AF. This statistical technique weighs individual studies by sample size and variance (both within- and between-study variance) and yields a pooled point estimate of OR and a 95% confidence interval (CI). The DerSimonian and Laird technique was considered an appropriate pooling technique because of the relative heterogeneity of the source population in each study. Subgroup analysis by study design was performed. We also evaluated the presence of heterogeneity across trials by using the I^2 ^statistic, which quantifies the percentage of variability that can be attributed to between-study differences [28]. Sensitivity analysis was performed to examine the effect of studies lacking information on methodologic quality on the pooled estimates. To assess the potential for publication bias, we performed the Begg's and the Egger's tests and constructed funnel plots to visualize possible asymmetry [29]. Due to the limited number of studies, no further subgroup analysis or meta-regression was considered. All statistical analyses were performed in Stata 10 (Stata Corp, College Station, TX, USA). We followed the Meta-analyses of Observational Studies in Epidemiology guidelines [30] in the report of this meta-analysis.

## Results

### Study characteristics and quality

**Figure 2:**
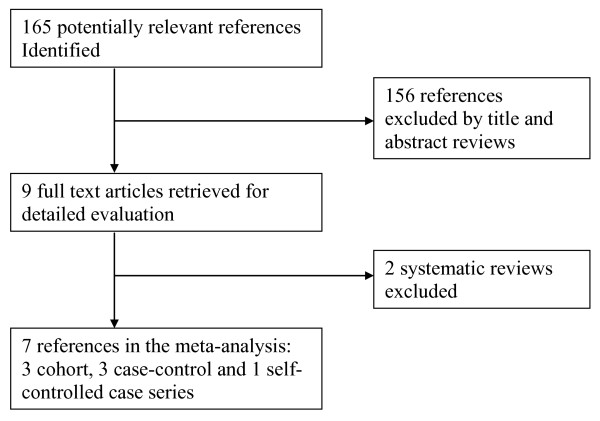
**Selection of studies included in the analysis**.

The electronic search retrieved 160 potentially relevant references (Figure 2). A manual search of bibliographies of relevant review papers and the published abstracts of the annual meetings of the American College of Rheumatology and the International Conference on Pharmacoepidemiology (2008 to 2009) identified five additional references. On initial screening, 156 of 165 were excluded based on titles and abstracts. Nine full-text articles were subsequently retrieved for detailed evaluation. Of those, seven studies - three cohort, three case-control, and one self-controlled case series [7,14-19] - representing data from 266,761 patients were finally included in the meta-analysis and two systematic reviews [20,21] were excluded. The characteristics of the included studies and of their participants are summarized in Tables 1 and 2. The self-controlled case series study by Grosso and colleagues [16] was considered as a case-control study for analysis.

One study was completed in Taiwan [17], and all others used population-based data from either North America [7,15,18] or Europe [14,16,19]. The number of subjects in each study ranged from 1,685 to 81,640. The majority of study subjects were aged over 70 years. Two studies compared bisphosphonate users with raloxifene users [17,18] and the rest compared bisphosphonate users with non-users [7,14-16,19]. One study [15] included zoledronic acid and the others only considered oral bisphosphonates. In the study by Levesque and colleagues [18], the risk of AF specific to alendronate, risedronate, and etidronate was reported; however, only the risk of AF among alendronate users was included in our meta-analysis because the majority of the included studies focused on either alendronate or a combination of different bisphosphonates as a drug class. Results from two different patient cohorts were available in the study by Bunch and colleagues [15].

Most studies were of moderate to high quality although only three studies defined the outcome of AF based on a combination of diagnosis codes and clinical records [7,14,15]. Quality of the study by Levesque and colleagues [18] was not assessed because it was only available as an abstract.

### Bisphosphonates and atrial fibrillation

**Figure 3:**
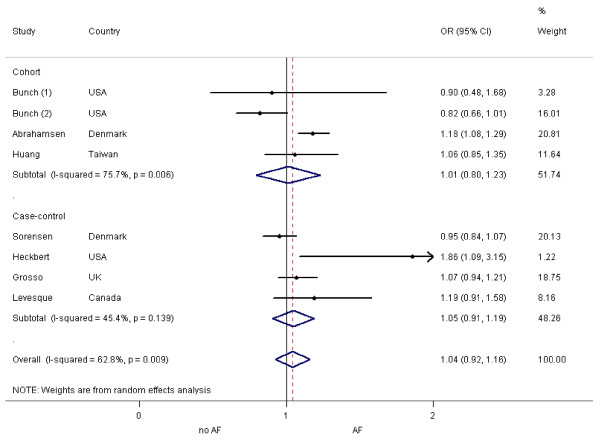
**Random effects analysis of the studies for the association between AF and bisphosphonate exposure, adjusted for known risk factors**. Points (dot) and overall (diamond) estimates are given as odds ratios (OR) with 95% confidence interval (CI) (horizontal bar), AF, atrial fibrillation.

The overall pooled estimate of multivariate OR for AF based on eight patient cohorts from seven studies adjusting for known AF risk factors was 1.04 (95% CI = 0.92 to 1.16; Figure 3). Moderate heterogeneity was noted (I^2 ^= 62.8%, *P *= 0.009). Our subgroup analysis stratified by study design yielded similar results. The pooled estimate of multivariate OR based on three cohort studies [14,15,17] was 1.01 (95% CI = 0.80 to 1.23) with moderate-to-severe heterogeneity (I^2 ^= 75.7%, *P *= 0.01). The pooled estimate of multivariate OR based on four case-control studies [7,16,18,19] was 1.05 (95% CI = 0.91 to 1.19) with moderate heterogeneity (I^2 ^= 45.4%, *P *= 0.14).

Sensitivity analysis was carried out by excluding the study by Levesque and colleagues [18], of which quality was not known. The pooled estimate of multivariate OR remained almost the same (1.03; 95% CI = 0.90 to 1.15).

### Publication bias assessment

**Figure 4:**
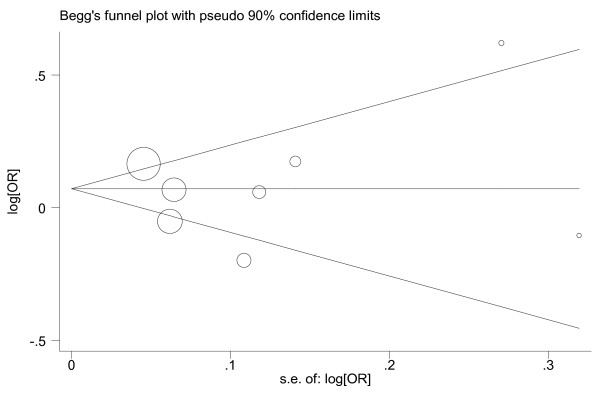
**Begg's funnel plot for publication bias assessment**. The size of circle represents the weight of each study in our analysis. OR, odds ratio; se, standard error.

The funnel plot was visually examined and did not reveal any obvious asymmetry (Figure 4). There was no statistical evidence of publication bias among the included studies by using Egger's (*P *= 0.90) and Begg's (*P *= 1.00) tests.

## Discussion

Although a few plausible mechanisms exist for the association between the use of bisphosphonates and AF -- electrolyte imbalance such as hypocalcemia or bisphosphonate-induced inflammation affecting atrial remodeling and fibrosis [31-34] - our meta-analysis of non-experimental studies found no significant association between bisphosphonate exposure and AF.

It is possible that our result was negative because mild or asymptomatic AF cases were missed in large non-experimental studies, because the majority of the included studies defined outcomes mainly with diagnosis codes. Also, as there is only a small fraction of patients exposed to intravenous forms of bisphosphonates such as ibandronate or zoledronic acid in our meta-analysis, it still remains uncertain whether the risk of AF increases with intravenous bisphosphonates. Our study is consistent with the FDA's safety review and supported by two previously published meta-analyses [20,21]. In the previous meta-analysis of four randomized clinical trials based on total 519 (230 serious, 289 non-serious) AF events among 26,352 patients [20], a significant association was observed between the risk of serious AF events and use of bisphosphonates (OR = 1.47; 95% CI = 1.01 to 2.14), but no significant association was noted for any AF events (OR = 1.14, 95% CI = 0.96 to 1.36). The elevated risk of serious AF might have been overestimated as two of the four clinical trials [5,6,35,36] were performed to study the effect of monthly zoledronic acid infusion on osteoporosis [5,36]. Another meta-analysis by Mak and colleagues [21] showed no evidence of an increased risk of AF related to bisphosphonate use in their meta-analysis of four randomized clinical trials [5,6,36,37] and three non-experimental studies (one cohort and two case-control studies) [7,14,19]. The pooled estimate of crude ORs based on three non-experimental studies [7,14,19] was 1.25 (95% CI = 0.98 to 1.73). Our study included four additional studies [15-18] that were not analyzed in any of previous meta-analyses and yielded similar results.

Several other limitations of our study should be noted. Due to a relatively small number of studies included in our analysis, we were unable to examine the risk of AF relative to the type or dose of bisphosphonates. Non-experimental studies are subject to measured and unmeasured confounding and other biases. Thus, our meta-analysis may suffer these limitations as well. Not every study included in our meta-analysis was adjusted for all the potential risk factors of AF, as observed in Tables 1 and 2. As raw data of individual studies were not available to us, we pooled the multivariate, or adjusted, ORs from the final model of each study. In addition, significant heterogeneity between studies was noted, as often expected in meta-analyses of observational studies [38,39]. In order to account for both within- and between-study variance, we used a random effects model for our analysis. Potential sources of heterogeneity between the studies include variations in the study size, patient characteristics (age, gender, geographic location, underlying comorbidities and use of other drugs), type of bisphosphonates, exposure and outcome ascertainment methods, and the study quality. Due to the limited number of the included studies in our analysis, we could not further identify causes of heterogeneity using the meta-regression technique. Although all meta-analyses are inherently vulnerable to publication bias, we attempted to minimize this bias by searching two major electronic databases with no geographic restriction. Three different statistical tests examined the issue of publication bias and revealed no statistical evidence for significant publication bias.

Our study also has several important strengths. This meta-analysis is up to date and comprehensive including seven large epidemiologic studies conducted in various countries [7,14-19]. We assessed the quality of individual studies using the Newcastle-Ottawa Scale [23]. The majority of the included studies had adequate sample sizes, follow-up lengths (applicable for cohort studies only), and adjustments for known AF risk factors. Our pooled estimates are based on multivariate ORs of individual studies adjusting for a number of known AF risk factors. We furthermore provided a subgroup analysis of the studies by study design and yielded a similar result.

## Conclusions

Our meta-analysis of published non-experimental studies suggests that there is no increased risk of AF associated with the use of bisphosphonates for osteoporosis. Physicians should be vigilant for all potential drug-associated toxicities, although the use of oral bisphosphonates probably does not increase a risk of AF, independent of known risk factors for AF. Future research studying a risk associated with intravenous bisphosphonates or a dose-response relation might provide further information.

## Abbreviations

AF: atrial fibrillation; CI: confidence interval; FDA: Food and Drug Administration; OR: odds ratio.

## Competing interests

SYK, MJK, and SMC declare that they have no competing interests. DHS received research grants from Merck & Co., Inc. Novartis, and Amgen, Inc.

## Authors' contributions

All authors participated in the study conception. SYK, SMC, and DHS participated in the study design and data interpretation. SYK and MJK participated in data acquisition and analysis. All authors participated in manuscript preparation and revision. All authors read and approved the final manuscript.
